# Uniaxial ferroelectric quantum criticality in multiferroic hexaferrites BaFe_12_O_19_ and SrFe_12_O_19_

**DOI:** 10.1038/srep25724

**Published:** 2016-05-17

**Authors:** S. E. Rowley, Yi-Sheng Chai, Shi-Peng Shen, Young Sun, A. T. Jones, B. E. Watts, J. F. Scott

**Affiliations:** 1 Cavendish Laboratory, Cambridge University, J. J. Thomson Avenue, Cambridge, CB3 0HE, United Kingdom; 2 Centro Brasileiro de Pesquisas Físicas, Rua Dr Xavier Sigaud 150, Urca, Rio de Janeiro, 22290-180, Brazil; 3 Beijing National Laboratory for Condensed Matter Physics, Institute of Physics, Chinese Academy of Sciences, Beijing 100190, China; 4 IMEM-CNR, Parco Area delle Scienze 37/A, 43124 Parma, Italy; 5 Schools of Chemistry and of Physics and Astronomy, St. Andrews University, St. Andrews, Fife, KY16 9ST, United Kingdom

## Abstract

BaFe_12_O_19_ is a popular M-type hexaferrite with a Néel temperature of 720 K and is of enormous commercial value ($3 billion/year). It is an incipient ferroelectric with an expected ferroelectric phase transition extrapolated to lie at 6 K but suppressed due to quantum fluctuations. The theory of quantum criticality for such uniaxial ferroelectrics predicts that the temperature dependence of the electric susceptibility χ diverges as 1/*T*^3^, in contrast to the 1/*T*^2^ dependence found in pseudo-cubic materials such as SrTiO_3_ or KTaO_3_. In this paper we present evidence of the susceptibility varying as 1/*T*^3^, i.e. with a critical exponent γ = 3. In general γ = (*d* + *z* – 2)/*z*, where the dynamical exponent for a ferroelectric *z* = 1 and the dimension is increased by 1 from *d*_*eff*_ = 3 + *z* to *d*_*eff*_ = 4 + *z* due to the effect of long-range dipole interactions in uniaxial as opposed to multiaxial ferroelectrics. The electric susceptibility of the incipient ferroelectric SrFe_12_O_19_, which is slightly further from the quantum phase transition is also found to vary as 1/*T*^3^.

Hexagonal ferrites are the most common magnetic materials with 90% of the $4 billion world market. 300,000 tons of hexagonal BaFe_12_O_19_ are produced every year, which corresponds to 50 grams for every person on Earth^1^,^2^. Primary uses are magnetic credit cards, bar codes, and small motors, as well as low-loss cheap microwave devices. In 2011 Fujifilm produced a barium hexaferrite-based tape with a memory of five terabytes − the equivalent of eight million books. At present this material has a new aspect of fundamental interest – it is nearly ferroelectric as the temperature approaches absolute zero. Incipient ferroelectrics at low temperature, i.e. materials close to a ferroelectric quantum phase transition, are expected to be important for a wide range of advanced material applications including for example, electro-caloric refrigeration, quantum memory devices, and cryogenic electronic switches, as their properties can be readily controlled by voltage gates and strains.

Very recently we examined^3^ the quantum criticality of uniaxial ferroelectric tris-sarcosine calcium chloride-bromide (TSCC:Br) and found that the low-temperature dielectric constant diverged with temperature as 1/*T*^2^, as in pseudo-cubic compounds such as strontium titanate^4^ and in contrast to the inverse cubic dependence first predicted by Khmelnitskii and Shneerson^5^, and were able to show that this paradox arises from the ultra-weak ferroelectric dipoles in that material. Here we report the study of a second uniaxial paraelectric, M-type barium hexaferrite, near its ferroelectric quantum phase transition, which is a strongly displacive system, with an A_2u_ symmetry soft mode frequency decreasing at the zone centre to 42 cm^−1^ as *T* goes to zero^6^. The only other low-*T* multiferroic studied in detail previously is EuTiO_3_^7^ which appears to be slightly too far from the critical point to manifest quantum critical behaviour.

There has been some controversy concerning ferroelectricity in this family of M-type hexaferrites: polarization-electric field hysteresis loops *P*(*E*) of SrFe_12_O_19_ at 300 K were published by Tan and Wang^8^,^9^, and there is also a recent theoretical paper^10^ by Wang and Xiang that predicts a paraelectric to antiferroelectric phase transition for BaFe_12_O_19_ at about 3.0 K. In this context it is important to note that SrFe_12_O_19_ and (Ba,Sr)Fe_12_O_19_ are n-type semiconductors^11^ with bandgaps at approximately E_g_ = 0.63 eV and rather heavy electrons and holes: m(light e) = 5.4 m_e_; m(heavy e) = 15.9 m_e_; m(light h) = 10.2 m_e_; m(heavy h) = 36.2 m_e_ and highly anisotropic conductivity, so it is important to discriminate between true ferroelectric hysteresis and leakage current artefacts. For electric fields applied normal to the c-axis, the electrical conductivity is ca. 50× greater than along c, which will create strong leakage currents. The present work and ref. 12 show that these suggested ferroelectric transitions do not occur at finite temperatures and that BaFe_12_O_19_ retains its paraelectric P6_3_/mmc symmetry (D_6_ _h_) down to zero temperature. When fitting the inverse susceptibility 1/χ_*E*_ to a Curie-Weiss law at higher temperatures (the linear part of the curve), an extrapolation to 1/χ_*E*_ = 0 gives an expected Curie temperature, like that in SrTiO_3_ or KTaO_3_, at ca. 6 K (similar to the 35 K value in SrTiO_3_). However the anticipated ferroelectric state does not stabilize and is suppressed by quantum fluctuations (analogous to the freezing temperature of liquid helium) resulting in a paraelectric ground state with quantum critical fluctuations. The proximity to the quantum critical point is evident from a rapidly rising dielectric susceptibility as the temperature is lowered and a soft A_2u_-symmetry *q* = 0 long wavelength phonon mode with a frequency that decreases to 42 cm^−1^ as *T* approaches zero^6^; we designate this frequency gap (minimum in the transverse-optical phonon frequency in the low temperature limit at *q* = 0) as Δ. A good review of work on SrFe_12_O_19_ was given this year by Hilczer *et al*.^13^. From a magnetic point of view Ba-hexaferrite is unusual in that although all 24 spins per primitive unit cell (two formula groups) are Fe^+3^, it is a ferrimagnet with 8 spins up (at tetrahedral, octahedral, and five-fold coordinated sites) and 16 down (all at octahedral sites), as shown in Fig. 1a. This produces a strong ferromagnetic moment, unlike weak canted antiferromagnets (it is often termed a Lieb-Mattis ferrimagnet^14^).

M-type hexaferrite single crystals were prepared by the flux method. The single-crystal x-ray diffraction (XRD) patterns at room temperature shown in Fig. 1c suggest that our samples are single-phase M-type with *c* = 23.18 Å for BaFe_12_O_19_ and 23.04 Å for SrFe_12_O_19_, respectively, agreeing with the original 1959 single-crystal value of Brixner^15^,^16^. The structure is not completely agreed upon in the literature: Ganapathi *et al*.^17^ report a tripled unit cell along the a-axis for flux-grown crystals like those in the present work; this differs from the original structural determination^15^ with *a* = 5.895 Å. However, this is unimportant for the present study since the ferroelectric properties are thought to involve only symmetry changes along the c-axis.

In a previously published paper^12^ we found that M-type ferrimagnetic hexaferrites (Ba,Sr)Fe_12_O_19_ are a new family of magnetic quantum paraelectrics along the *c*-axis only. This preservation of c-axis six-fold symmetry is compatible with the A_2u_-symmetry soft mode reported from the Rostov group, which retains the hexagonal symmetry. The resulting symmetry of the crystal, were it to undergo a transition into a ferroelectric phase, is therefore probably C_6v_ point group symmetry and P6_3_/mc space group. Because there is no change in hexagonal crystal class, this transition would be purely ferroelectric and not ferroelastic^18^, with no hysteresis in its stress-strain relationship. That may be important with regard to descriptions of the system close to quantum criticality, implying that no elastic order parameter is a conjugate force.

As shown in Fig. 1b, M-type hexaferrite exhibits a new mechanism for local electric dipoles based on the magnetic Fe^3+^ (3d^5^) ion, violating the d^0^ rule of Nicola Hill^19^. The competition between the long-range Coulomb interaction and short-range Pauli repulsion in a FeO_5_ bipyramid with proper lattice parameters would favour an off-centre displacement of Fe^3+^ that induces a local electric dipole. Such local dipoles cannot order down to the lowest temperatures in the specimens we measured but ferroelectric ground states may be reached perhaps via tuning with strains, chemical substitution or by varying the lattice density.

## Results and Discussion

**Figure 2:**
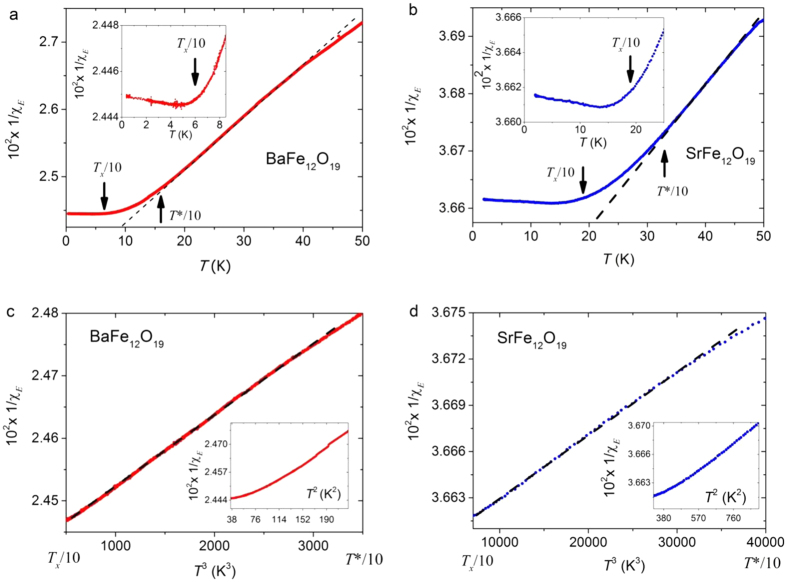
Dielectric susceptibility measurements along the c-axis in Ba and Sr M-type hexaferrites. The main figures in (**a**,**b**) show the temperature depence of the inverse electric susceptibility 1/χ_*E*_ for BaFe_12_O_19_ and SrFe_12_O_19_ respectively. The classical Curie-Weiss like behaviour (linear part of the curve) at higher temperaures crosses over to a different form below the characteristic temperature scale T^*^/10 due to the proximity of a ferroelectric quantum phase transition. The insets in (**a,b**) show a magnification of the low temperature region of 1/χ_*E*_ against temperature in which anomolous upturns are observed below the temperature scale *T*_x_/10. In (**c,d**) the inverse electric susceptibily is plotted against *T*^3^ over the range of temperatures between the anomolous upturn at low *T* and the classical Curie-Weiss regime at high *T*, i.e. between *T*_*x*_/10 and T^*^/10 as explained in the text. This is between 6 K and 15 K for BaFe_12_O_19_ in (**c**) and between 20 K and 35 K for SrFe_12_O_19_ in (**d**). The dashed straight lines are guides to the eye. The lower insets in (**c,d**) confirm that no *T*^2^ dependence of 1/χ_*E*_ fits the data which would be expected for a multi-axial as opposed to a uniaxial ferroelectric quantum critical system. Recent attempts^20^ by others to fit data over a wide temperature range to a single exponent are in our opinion not reliable tests.

Our low temperature inverse electric susceptibility data 1/χ_*E*_, (related to the measured dielectric constant ε by χ_*E*_ = ε − 1), are shown in Fig. 2. The measurements were obtained with a pumped helium-3 cryostat for both cooling and heating cycles, typically at rates of 10 mK/minute (overnight runs). Here we see that below approximately 6 K in BaFe_12_O_19_ and 20 K in SrFe_12_O_19_ there is a non-monotonic dependence; we have previously reported such effects in SrTiO_3_, KTaO_3_^4^, and tris-sarcosine calcium chloride (TSCC)^3^ and shown quantitatively without adjustable parameters in the former cases that they arise from acoustic phonon coupling (electrostriction). Similarly such behaviour may occur from coupling of the polarization order-parameter field with any other auxiliary filed such as the magnetisation (magneto-capacitance or indirectly via magneto-striction). In SrTiO_3_ and KTaO_3_ the upturn in the inverse susceptibly, as determined by measurements and theory without adjustable parameters, occur when *T* is less than 10% of *T*_*x*_ where *T*_*x*_ is the temperature scale associated with the soft transverse-optical phonon frequency at the zone centre, 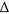, in the zero temperature limit, i.e. 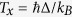. This means that we can attempt to fit the dielectric susceptibility data only for *T* > 0.1*T*_*x*_ to a quantum criticality model in the absence of magneto-electric or electrostrictive coupling terms, the parameters for which are not currently available for our samples. 0.1*T*_*x*_ = 6 K for BaFe_12_O_19_ as determined from measurements^6^, and estimated to be 20 K in SrFe_12_O_19_ which is further away from the quantum phase transition. Note that precisely at a ferroelectric quantum critical point, the frequency gap Δ vanishes and both *T*_*x*_ and the Curie temperature *T*_*C*_ are exactly zero. This means that such upturns only exist in samples with paraelectric ground states some distance away, but close to, the quantum phase transition. Another cross-over temperature exists for the upper temperature limit for any power-law exponent: In measurements and theory in SrTiO_3_ and KTaO_3_ we found that a single quantum critical exponent extends up to ca. 10% of the characteristic temperature T^*^. T^*^ is analogous to the Debye temperature but of the soft (critical) transverse-optical phonon mode and is given by 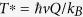 where *v* is the gradient of the frequency with respect to wave-vector *q* at low temperatures from a dispersion of the form 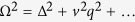 in the limit that Δ goes to zero, and *Q* is the value of *q* at the Brillouin zone boundary. It can also be estimated from 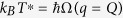 where 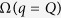 is the measured value of the transverse-optic mode frequency in the low temperature limit at the Brillouin zone boundary, *q* = *Q*. The soft mode dispersion in BaFe_12_O_19_ has not been measured, but based upon its frequency at *q* = 0 and the heavy masses in BaFe_12_O_19_, we estimate T^*^ to be approximately 150 K. Therefore in Fig. 2c we fit the measured dielectric susceptibility χ_*E*_(*T*) over the range 6–15 K for BaFe_12_O_19_, i.e. between the lower and upper crossover temperatures 0.1*T*_*x*_ and 0.1T^*^ respectively. Figure 2c shows that 1/χ_*E*_ varies as *T*^3^ for several thousand data points in the region, thus supporting the theory of Khmelnitskii and Shneerson^5^ of quantum criticality for a uniaxial ferroelectric. Above 10% of T^*^ one expects the system to exhibit classical Curie-Weiss behaviour as observed in our data in Fig. 2a,b. We emphasize that the critical exponent is measured with unusual precision as γ = 3.0 +/− 0.1 – and certainly not close to γ = 2.0 for multiaxial quantum critical systems as highlighted in the lower insets in Fig. 2c,d – over thousands of data points – every 5 mK in the range). Conversely, none of the other systems we have studied in the past gave exponents near 3.0 over any temperature range^3^,^4^. High pressure work by which *T*_*c*_ may be brought up through *T* = 0 will be the subject of future work, but the results of dielectric measurements in the presence of “chemical pressure” obtained by replacing Ba-ions with smaller Sr-ions is shown in Fig. 2b,d, where a cubic temperature dependence agrees with the measured data in the range 20 K to 35 K for SrFe_12_O_19_.

Hexaferrites are important and well known magnetic materials. Our work shows that at cryogenic temperatures the magnetic phase coexists with a quantum fluctuating electrical dipole phase coupled to the magnetic system. The quantum critical phase is evident by the fact that the equilibrium thermodynamic quantities such as the susceptibility, depend on both the static and dynamic (frequency-dependent) properties of the system, which results in a unity rise in the effective dimension. In the uniaxial materials investigated here, the cryogenic phase is particularly novel since apart from short-range interactions, long-range anisotropic electrical dipole interactions provide a further unity increase in the effective dimension to *d*_eff_ = 5 as detected by our high-precision dielectric measurements. The magnetic ordering temperature and magneto-electric behaviour of these materials may be tuned by chemical substitution realising a magnetic quantum phase transition and quantum glassy or relaxor type phenomena in different parts of the phase diagram. A detailed study of the frequency dependence of the dielectric and magnetic properties in these chemically or pressure tuned hexaferrites is likely to be the subject of promising future work.

## Methods

M-type hexaferrite single crystals were prepared by the flux method. The raw powders of BaCO_3_ (SrCO_3_), Fe_2_O_3_, and fluxing agent Na_2_CO_3_ were weighed in the molar ratio 10.53% : 26.3% : 63.17% and were well mixed. The mixed raw powder was put in a Pt crucible and heated to 1250 °C for 24 h in air, then cooled down to 1100 °C at a rate of 3 °C/min and finally quenched to room temperature. The samples (ca. 2 mm on a side) were characterized by single-crystal x-ray diffraction at room temperature by using a Rigaku X-ray diffractometer.

The dielectric measurements were carried out in a pumped helium-3 cryostat at temperatures as low as 0.3 K. Silver paste was painted on the surfaces (ab plane) of a thin plate of each crystal and an Andeen-Hagerling or Agilent 4980 A LCR meter was used to measure the dielectric susceptibility at frequencies typically in the range 1 kHz to 100 kHz.

## Additional Information

**How to cite this article**: Rowley, S. E. *et al*. Uniaxial ferroelectric quantum criticality in multiferroic hexaferrites BaFe_12_O_19_ and SrFe_12_O_19_. *Sci. Rep.* **6**, 25724; doi: 10.1038/srep25724 (2016).

## Figures and Tables

**Figure 1:**
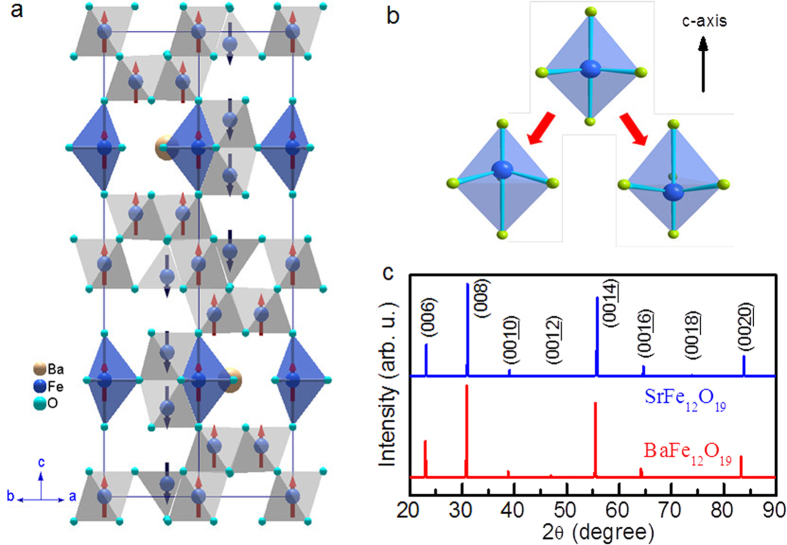
Crystal and multiferroic structures of M-type hexaferrites. (**a**) The crystal and magnetic structures of M-type Ba- and Sr- hexaferrites. The arrows represent the magnetic moments of Fe^3+^ ions. (**b**) The off-equator displacements of Fe^3+^ in the FeO_5_ bypyramidal sites induce uniaxial electric dipoles along *c* axis. Quantum fluctuations between two 4e sites prevent the onset of long-range ferroelectric ordering down to the lowest temperature. (**c**) The single-crystal x-ray diffraction patterns at room temperature of prepared BaFe_12_O_19_ and SrFe_12_O_19_ crystals.

## References

[b1] Pullar R. Hexagonal ferrites: A review of the synthesis, properties and applications of hexaferrite ceramics. Prog. Mat. Sci. 57, 1191–1334 (2012).

[b2] Pullar R. Multiferroic and Magnetoelectric Hexagonal Ferrites, Springer Series in Materials Science (eds Saxena A. & Planes A.) Volume 198, Mesoscopic Phenomena in Multifunctional Materials: Synthesis, Characterization, Modelling and Applications (Heidelberg, 2014).

[b3] Rowley S. . Quantum criticality in a uniaxial organic ferroelectric. J. Phys. Cond. Matt. 27, 395901 (2015). 10.1088/0953-8984/27/39/395901 26360383

[b4] Rowley S. . Ferroelectric Quantum Criticality. Nat. Phys. 10, 367–372 (2014).

[b5] Khmelnitskii D. E. & Shneerson V. L. Low-temperature displacement-type phase transition in crystals. Sov. Phys. Solid State 13, 687 (1971).

[b6] Mikheykin A. . Lattice anharmonicity and polar soft mode in ferrimagnetic M-type hexaferrite BaFe_12_O_19_ single crystal. Eur. J. Phys. B87, Art. No. 232 (2014).

[b7] Katsufuji T. & Takagi H. Coupling between magnetism and dielectric properties in quantum paraelectric EuTiO_3_. Phys. Rev. B64, 054415 (2001).

[b8] Tan G. & Wang M. Multiferroic PbFe_12_O_19_ ceramics. J. Electroceram. 26, 170–174 (2011).

[b9] Tan G. & Wang M. Structure and multiferroic properties of barium hexaferrite ceramics. J. Magn. Magn. Mat. 327, 87–90 (2013).

[b10] Wang P. & Xiang H. Room-Temperature Ferrimagnet with Frustrated Antiferroelectricity: Promising Candidate Toward Multiple-State Memory. Phys. Rev. X4, 011035 (2014).

[b11] Fang C., Kools F., Metselaar R., de With G. & de Groot R. Magnetic and electronic properties of strontium hexaferrite SrFe_12_O_19_ from first-principles calculations. J. Phys. Cond. Mat. 15, 6229–6237 (2003).

[b12] Shen S. . Magnetic-ion-induced displacive electric polarization in FeO_5_ bipyramidal units of (Ba,Sr)Fe_12_O_19_ hexaferrites. Phys. Rev. B90, 180404R (2014).

[b13] Hilczer A. . Effect of thermal treatment on magnetic and dielectric response of SrM hexaferrites obtained by hydrothermal synthesis. Phase Transitions 87, 938–952 (2014).

[b14] Lieb E. & Mattis D. Ordering energy levels of interacting spin systems. J. Math. Phys. 3, 749–756 (1962).

[b15] Brixner L. Preparation of the ferrites BaFe_12_O_19_ and SrFe_12_O_19_ in transparent form. J. Am. Chem. Soc. 81, 3841–3843 (1959).

[b16] Townes W., Fang J. & Perrotta A. Crystal structure and refinement of ferrimagnetic barium ferrite BaFe_12_O_19_, Z. Krist. Kristallgeom. Kristallphys 125, 437–445 (1967).

[b17] Ganapathi L., Gopalakrishnan G. & Rao C. Barium hexaferrite (M-phase) exhibiting superstructure. Mat. Res. Bull. 19, 669–672 (1984).

[b18] Toledano J. & Ferroelasticity A. Telecommunications 29, 249–270 (1974).

[b19] Hill N. Why are there so few magnetic ferroelectrics? J. Phys. Chem. B104, 6694–6709 (2000).

[b20] Cao H. . High pressure floating zone growth and structural properties of ferrimagnetic quantum paraelectric BaFe_12_O_19_. APL Mater. 3, 062512 (2015).

